# Dynamics of the Glycophorin A Dimer in Membranes of Native-Like Composition Uncovered by Coarse-Grained Molecular Dynamics Simulations

**DOI:** 10.1371/journal.pone.0133999

**Published:** 2015-07-29

**Authors:** Nadine Flinner, Enrico Schleiff

**Affiliations:** 1 Department of Biosciences, Molecular Cell Biology of Plants, Goethe University, Max von Laue Str. 9, 60438, Frankfurt/Main, Germany; 2 Cluster of Excellence Frankfurt, Goethe University, Max von Laue Str. 9, 60438, Frankfurt/Main, Germany; 3 Buchmann Institute of Molecular Life Sciences (BMLS), Goethe University, Max von Laue Str. 15, 60438, Frankfurt/Main, Germany; 4 Center of Membrane Proteomics, Goethe University, Max von Laue Str. 9, 60438 Frankfurt/Main, Germany; University of Minnesota, UNITED STATES

## Abstract

Membranes are central for cells as borders to the environment or intracellular organelle definition. They are composed of and harbor different molecules like various lipid species and sterols, and they are generally crowded with proteins. The membrane system is very dynamic and components show lateral, rotational and translational diffusion. The consequence of the latter is that phase separation can occur in membranes *in vivo* and *in vitro*. It was documented that molecular dynamics simulations of an idealized plasma membrane model result in formation of membrane areas where either saturated lipids and cholesterol (liquid-ordered character, L_o_) or unsaturated lipids (liquid-disordered character, L_d_) were enriched. Furthermore, current discussions favor the idea that proteins are sorted into the liquid-disordered phase of model membranes, but experimental support for the behavior of isolated proteins in native membranes is sparse. To gain insight into the protein behavior we built a model of the red blood cell membrane with integrated glycophorin A dimer. The sorting and the dynamics of the dimer were subsequently explored by coarse-grained molecular dynamics simulations. In addition, we inspected the impact of lipid head groups and the presence of cholesterol within the membrane on the dynamics of the dimer within the membrane. We observed that cholesterol is important for the formation of membrane areas with L_o_ and L_d_ character. Moreover, it is an important factor for the reproduction of the dynamic behavior of the protein found in its native environment. The protein dimer was exclusively sorted into the domain of L_d_ character in the model red blood cell plasma membrane. Therefore, we present structural information on the glycophorin A dimer distribution in the plasma membrane in the absence of other factors like e.g. lipid anchors in a coarse grain resolution.

## Introduction

Plasma membranes surround living cells and consist mainly of a bilayer composed of lipids, sterols and transmembrane proteins. The major lipids of the mammalian plasma membrane are phospho- and sphingolipids, and also contain a substantial amount of the sterol cholesterol [1,2]. The most common lipids are the phospholipids phosphatidylcholine (PC), phosphatidylethanolamine (PE), phosphatidylserine (PS) and the sphingolipid sphingomyelin (SM) that also carries a phosphocholine head group [2]. The two membrane leaflets have a distinct composition: PS and PE are pumped by the aminophospholipid translocase to the inner leaflet of the membrane [3], while SM is exclusively localized in the outer leaflet [4]. Within one leaflet lipids undergo rotational diffusion (rotation around the own axis), and lateral diffusion along the membrane plane [5]. The lateral diffusion coefficient of lipids is in the range of 10^−7^ to 10^−8^ cm^2^s^-1^, while the diffusion coefficient for transmembrane proteins is with ~10^−9^ cm^2^s^-1^ one magnitude lower [6]. Trans bilayer movement of lipids—also called flip-flop—was reported as well, however, the diffusion between the two leaflets is mostly assisted by proteins [7].

As a consequence of the diffusion of its components membranes are not homogenous and different domains with distinct lipid, sterol and protein composition exist [8]. One example for such domains in the plasma membrane are the small membrane rafts, which are only 10–200 nm in size and are highly dynamic structures [9]. They are enriched in sphingolipids, cholesterol and proteins, which are linked to membrane trafficking and signaling [10].

In three component model membranes composed of cholesterol, a saturated and an unsaturated phospholipid species, phase separation was determined by NMR and fluorescence microscopy [11]. This phenomenon could be reproduced by the theoretical approach of coarse-grained (CG) molecular dynamics (MD) simulations [12]. Membrane domains in the liquid-ordered (L_o_) state containing saturated lipids and cholesterol, and the liquid disordered (L_d_) state with unsaturated lipids are formed in such model membranes [11,12]. By confocal microscopy and CG MD simulations it was observed that transmembrane α-helix model peptides (WALP peptide) are sorted into the membrane domain with L_d_ state independently of the hydrophobic length of the α-helix of the model peptide [13]. Furthermore, CG MD simulations showed that the sorting of transmembrane peptides into the different membrane domains is modified by the presence of a lipid anchor at the protein and somewhat depends on the lipid species used for modeling the membrane [14].

The importance of understanding the lipid distribution in different membrane domains arises from the fact that transmembrane protein function is influenced by the membrane environment: Some proteins have a lipid non-covalently bound to their surface, which is required to stabilize the structure or is important for their enzymatic function [15]. Furthermore, the activity of some transmembrane proteins is regulated by the hydrophobic thickness or the intrinsic lipid curvature of the membrane [16]. Moreover, some proteins even sense the state of the membrane to transmit this information into the regulatory network of the cell [2,17].

Since interactions between lipid and proteins are essential for membrane function and integrity several mechanisms are known to avoid unfavorable conditions. For example, a hydrophobic mismatch between the transmembrane domain of a protein and the membrane is energetically unfavorable [18,19] and is often balanced by adopting the thickness of the membrane by stretching or compressing the fatty acid acyl chains [20,21]. Alternatively, protein tilting is observed to reduce the mismatch for proteins with a too long hydrophobic length [22]. Aggregation of proteins is also a common mechanism to reduce the energy penalty for the mismatch between the membrane and the protein [23].

Recently, a model of an idealized plasma membrane was analyzed by CG MD [24]. This study confirmed that cholesterol undergoes a flip-flop movement in the membrane of native-like composition and revealed that cholesterol resides more frequently in the outer leaflet. Remarkably, this study provided first evidence that macroscopic domain formation does not occur, but small membrane domains enriched in saturated or unsaturated lipids are formed [24].

To investigate the distribution of proteins in the membrane of native-like composition, we built a model of the red blood cell (RBC) plasma membrane and inserted a glycophorin A (GpA) dimer. GpA is one of the most abundant proteins in the RBC plasma membrane [25,26]. It contains a single transmembrane α-helix and its dimerization is mediated by a GxxxG motif within the transmembrane helix as shown by NMR structures [27,28]. Such a motif is common for dimerization of many transmembrane proteins [29]. We explored the importance of lipid species as well as of the presence of cholesterol for sorting and dynamics of the protein by simulations of various membrane forms. We document that the cholesterol and the fatty acid composition are the major determinants for sorting and dynamics of the protein. In contrast the head group composition within the membrane does not have a significant influence on the protein and membrane dynamics.

## Methods

### Simulation details

All simulations were performed using GROMACS v4.5.5 [30] and the coarse-grained MARTINI 2.2 force field [31–34]. Standard MARTINI parameters were used for generation of trajectories. For temperature coupling the v-rescale method (ref-t: 320 K, [35]) and for pressure coupling the parrinello-rahman method (semi-isotropic; tau-p: 12 ps; ref-p: 1 bar, [36]) was applied. The neighbor lists had a cutoff of 1.4 nm and were updated every 10^th^ time step. Non-bonded interactions were cut off at 1.2 nm (0.9 to 1.2 nm for Lennard-Jones potential and smooth switching from 0.0 to 1.2 nm for Coulomb potential, epsilon-r: 15) and a time step of 0.02 ps was used. Plain simulation times are reported in the manuscript, they were NOT multiplied by a factor of 4 to account for the speed-up caused by the CG procedure [31,32].

### Construction and composition of the membrane

The composition of the RBC plasma membrane was chosen based on 1 and is given in Table 1. Components with a frequency lower than 3% were not considered. Lipid parameters not accessible in the standard MARTINI lipid files were generated by shuffling fatty acid and head group building blocks, a method that was previously established [24]. To represent the different properties of the PE-pl (PE-based plasmalogen) linker, which is the only building block, which is not given in the lipid force field, with one fatty acid bound via an ether and one via an ester group. In general the Martini lipid force field provides chemical building blocks which are parameterized to reproduce different experimental measurements like e.g. the partitioning between water and an organic phase (e.g. hexadecane or octanol) [32], mimicking a membrane environment. Here methylformate (C-O-C = O) is modelled with a N_a_ bead type, which is also used for the linker of ester lipids, and methoxyethane (C-O-C_2_) is modelled with a N_o_ bead [32]. According to this assignment we modelled the ester with N_a_ and the ester with N_0_. All other parameters (bonded and non-bonded) are the same as for PE lipids.

**Table 1:** Starting composition of the model of the RBC membrane.

head group		PC		SM	PS	PE			PE-pl	
fatty acid	CHOL	16:0	16:0	16:0	18:0	16:0	18:0	18:0	16:0	18:0
		18:2	18:1	16:0	20:4	18:1	20:4	20:4	20:4	20:4
count inner	245	25	20	0	90	30	30	20	28	12
count outer	245	63	52	140	0	0	0	0	0	0

By scattering and atomistic MD simulations it is shown that the hydroxyl group of cholesterol prefers the interaction with the phosphate oxygen in membranes with ether lipids [37], while for membranes with ester lipids cholesterol prefers the interaction with the backbone ester carbonyls as shown by MD simulations [38]. This indicates that cholesterol is located nearer to the water interface and is less buried in the membrane. In line using the N_a_ bead type to represent ether lipids it could be observed that the interaction with the PO4 bead is enhanced in CG MD simulation in a model membrane containing ether phospholipids and cholesterol in comparison to a model membrane containing ether phospholipids and cholesterol. Furthermore cholesterol resides closer to the water interface in the membrane containing ether lipids (S1 Fig).

The membrane was constructed as follows: A membrane with 510 DOPC and 490 cholesterol, solvated by 10,000 CG water molecules was generated by a self-assembly simulation. To create the native-like membrane the DOPC molecules of the model membrane were randomly replaced with lipids present in a native membrane (Table 1), while it was ensured that the lipids with different head groups are located at the correct leaflet (PE, PE-pl and PS: inside, SM: outside; PC and CHOL: both). 90 water beads were exchanged with ion beads (NA+) to obtain a system with zero net charge. All membranes were energy minimized followed by an MD run with position-restrained lipids. Finally, the membrane was simulated for 10 μs to ensure lipid self-organization. Finally, the CG structure of the NMR structure of the GpA dimer [27] was inserted into the model membrane and the system was energy minimized followed by an MD run with position-restrained protein. Subsequently, ten CG MD simulations for 10 μs using different random seeds for velocity generation were performed without any position restraints.

For the system missing cholesterol (nFA+head) all cholesterol molecules were removed from the model of the RBC membrane including the protein. The system for the simulations, where the membrane contains only PC lipids with native fatty acids and cholesterol (nFA+CHOL), were generated by swapping all head groups back to PC and all ions were replaced with CG water molecules. The system for the simulations with a membrane containing only the native fatty acids (nFA) was derived from the nFA+CHOL system by deleting the cholesterol molecules. Subsequently, all three newly formed systems were energy minimized and simulated for a short MD run, where the protein dimer is position-restrained. The resulting lipid composition of the three systems is given in S1 Table.

Again, these systems were each simulated ten times with different random seeds.

### Analysis of trajectories

All trajectories were analyzed with different GROMACS [30] and YASARA [39] tools. In general, frames obtained every 50 ns were used for analysis. YASARA was used e.g. for system visualization and for readout of bead positions and determination of bead distances. For the construction of the density profile of the membrane the *g_density* tool was used. The leaflet assignment of cholesterol molecules was determined based in the position of its ROH bead (hydroxyl group) relative to the position of the lipid linker beads.

The analysis of visited interfaces of the GpA dimer was performed as described [40], in short: For each frame a contact map was constructed saving the occurring residue contacts. Based on these contact maps a distance matrix, using the Dice dissimilarity, was calculated to perform a clustering and to group the same interfaces. The frequency vectors obtained from the clustering of different conditions were compared using Pearson’s correlation coefficient.

The tool *g_rdf* was used to get distances of different hydration shells around the protein. A lipid was considered to be in the first/second/third hydration shell of the protein, if its distance to the protein is less than 0.658/0.918/1.178 nm according to the local minima of the radial distribution function. The obtained composition of these hydration shells was then compared to the total frequency of the lipid in the corresponding leaflet. Already within the first 0.5 μs the lipids which surround the protein were exchanged and differ between the ten simulations (S2 Fig). To determine the time a lipid was maximally bound to the protein (here frames obtained every 0.5 ns where used for analysis), a lipid was considered to be bound for the time where its distance was smaller than 0.658 nm. The 2D density maps of areas enriched or depleted in cholesterol, lipids with saturated and monounsaturated fatty acids and lipids with polyunsaturated fatty acids were generated using the *g_densmap* tool to monitor the density over a timespan in a window of 2 μs in the x-y plane of the simulation box. To visualize which areas co-localize the three maps were merged: In the merged 2D density map only those regions are indicated, which are enriched in a single molecule class. A region is counted as enriched, if the density in this point is at least as high as 2/3 of the maximal density of this molecule in the whole box. Regions enriched in cholesterol and lipids with saturated/monounsaturated fatty acids are indicated with a fourth color. The lateral diffusion is calculated using the *g_msd* tool, the resulting values are not corrected by a special factor.

## Results and Discussion

### Model of the RBC plasma membrane

**Fig 1:**
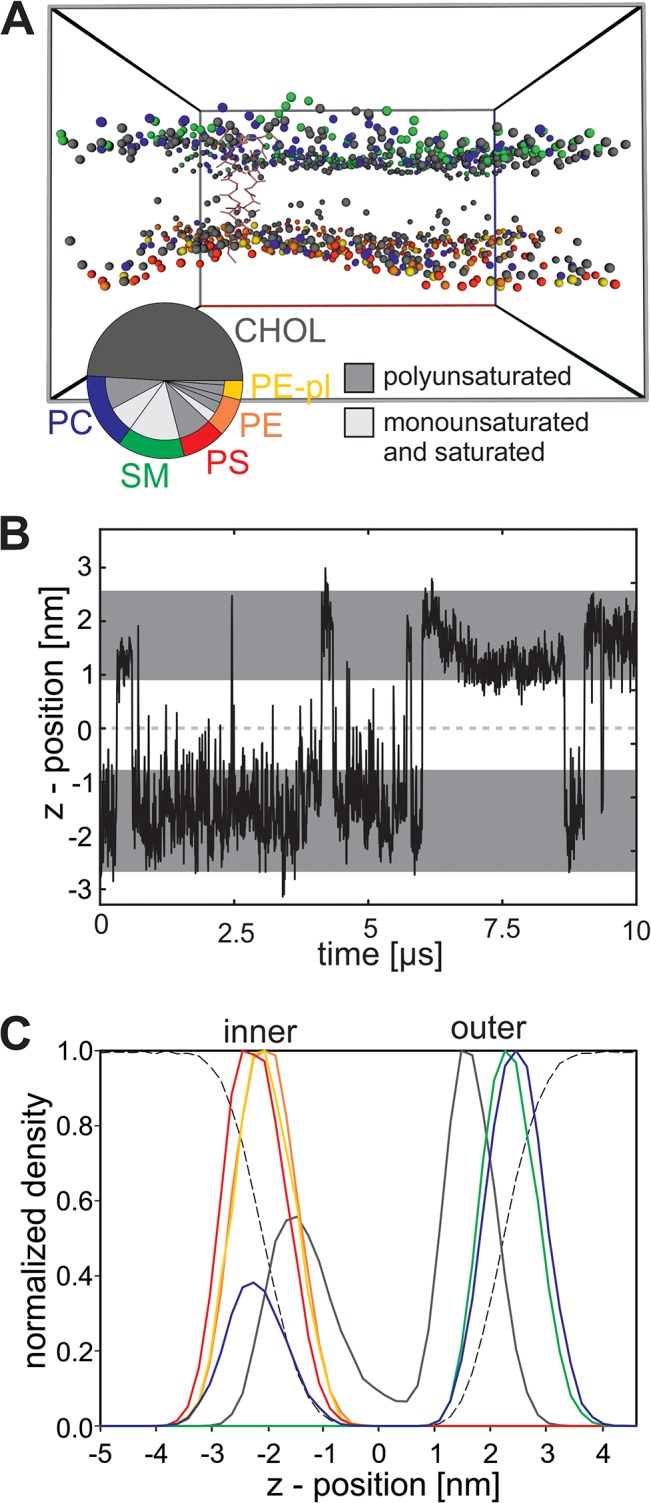
Model of the RBC plasma membrane. (A) The native membrane system in the simulation box after 10 μs simulation is shown. All water molecules are removed for clarity. The protein is shown in stick representation (rose). For lipids only one bead is displayed: For cholesterol (CHOL) the ROH bead (hydroxyl group(grey), for PC (blue), SM (green), PS (red), PE (orange) and PE-pl (yellow) one bead of the lipid linker (GL1 or AM1). The pie chart displays the composition of the native-like membrane. The outer ring corresponds to the head group and the inner ring to the fatty acid. Because cholesterol does not contain a classical fatty acid, only one ring is displayed. (B) Position of the ROH bead of a representative cholesterol molecule during the simulation of the native-like membrane. The dark grey areas indicate the position (average ± 2*standard derivation) of the lipid backbone (bead GL1 of PE 16:0/18:1 for the inner leaflet and bead AM1 of SM for the outer leaflet). (C) Density profile across the native-like membrane for one representative simulation. For all lipids the density of the outermost bead is shown (cholesterol: ROH; PC and SM: NC3; PS: CNO; PE and PE-pl: NH3). The color code is given in A.

We constructed a model of the RBC plasma membrane with an integrated GpA dimer (Fig 1A; Methods) to investigate the dynamics of the system and the components thereof. The model membrane contains cholesterol, PC, SM, PS, PE as well as PE-pl lipids with fatty acids, which are common for the corresponding head group in ratios according to Leidl and coworkers [1]. Minor lipid components (less than 3%), like for example phosphatidylinositol (PI), were not included into the model. The different lipids were asymmetrically distributed across the membrane according to the distribution in the native membrane [3,4]. Here, PE, PE-pl and PS were exclusively positioned in the inner leaflet, SM was incorporated exclusively into the outer leaflet and PC is present in both leaflets, but with a higher frequency in the outer leaflet (Table 1). At the beginning of the simulation cholesterol was evenly distributed across both leaflets. Due to the high frequency of flip-flop movement of cholesterol during the simulation time of 10 μs (Fig 1B), cholesterol is unevenly distributed between both leaflets as indicated by the density profile of the membrane (Fig 1C). On average the outer leaflet contains ~274 and the inner leaflet ~190 cholesterol molecules after equilibration, while the remaining cholesterol molecules are located in the hydrophobic core of the membrane. This imbalance of cholesterol distribution was also observed by Ingolfsson and coworkers [14]. In agreement the cholesterol flip-flop rates have a half-time of <1 s, as determined by experiments [41]. Thus it is possible that the flipping as determined in here using the Martini force field is may be to fast, although it agrees with former simulation studies.

Previously, the thickness of the hydrophobic core and the distance between the lipid head groups of the RBC plasma membrane was experimentally determined to be 2.5 nm and 4.8 nm, respectively [42]. These values are in good agreement with the distances extracted from the density profile of the modeled membrane. We determined an average distance of 2.9 nm between the hydroxyl beads (ROH) of cholesterol and of 4.5–4.9 nm between the head groups of the phospho- and sphingolipids (Fig 1C). Furthermore, the lateral diffusion of the lipids (2.05 * 10^−7^ cm^2^/s) and the protein (6.14 10^−8^ cm^2^/s) observed in the simulation is in good agreement with experimental values which also vary with one order of magnitude (lipid diffusion in natural membranes ~10^−8^ and diffusion for membrane proteins ~10^−9^; [6]). Taken together the simulated system reproduces experimentally determined values and thus, is valid to investigate the properties of the RBC plasma membrane and of the GpA dimer therein.

### Protein-protein interaction

**Fig 2:**
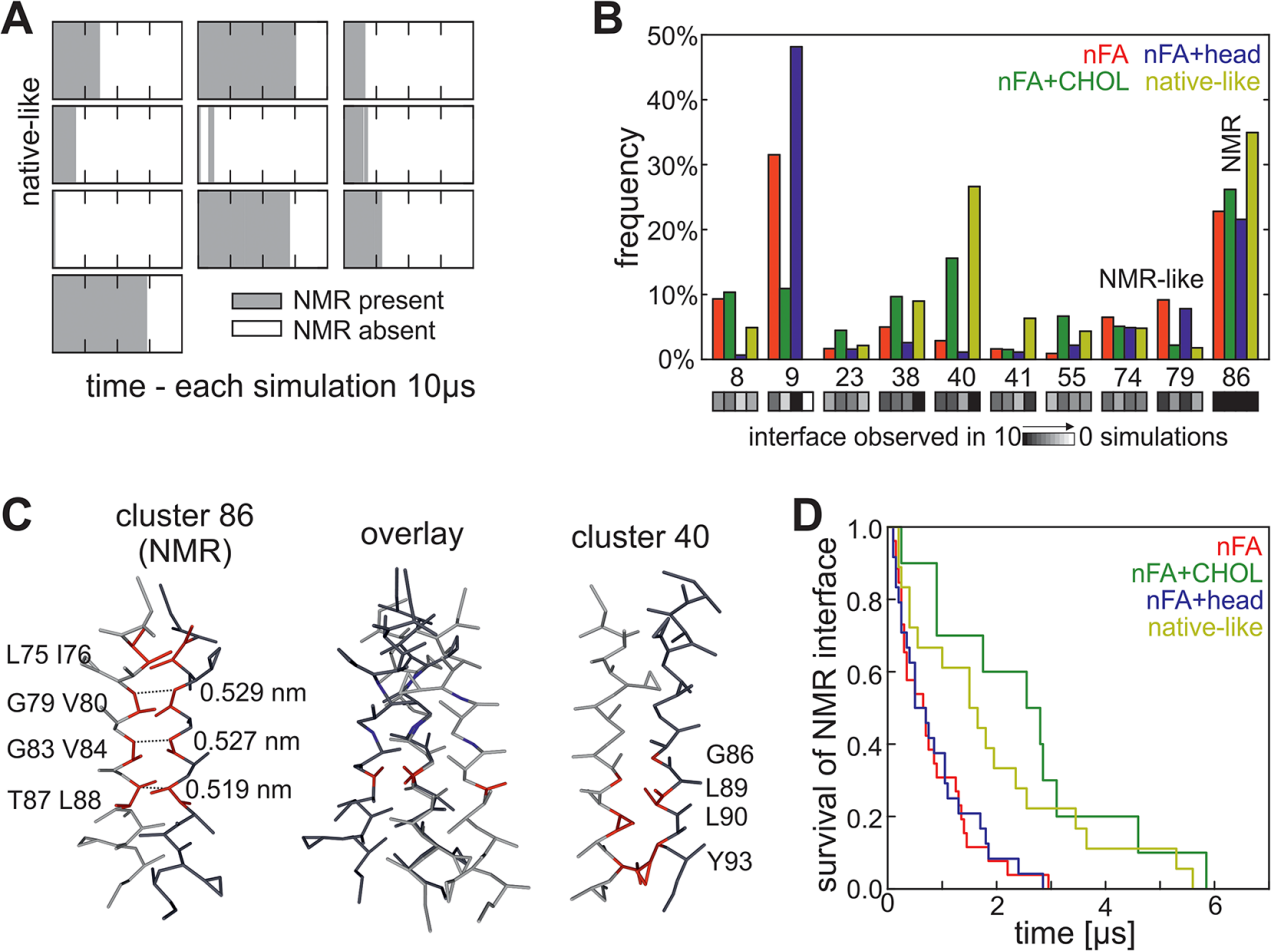
Dynamics of the protein in the RBC plasma membrane. (A) For all ten simulations of the model of the RBC plasma membrane (native-like) the presences of the NMR interface is plotted for each time point in grey. (B) Shown is the frequency of frequent interface clusters (frequency over 10% in all simulations) from all ten simulations per membrane (nFA (red), nFA+CHOL (green), nFA+head (blue), native-like (yellow)). The number of simulations, in which the corresponding interface was identified, is given on the bottom of each cluster (in grey scale). (C) Screenshot of representative structures from cluster 86 (left) and cluster 40 (right) and in the middle a structural alignment is shown. In the screenshot left and right the main interface residues are highlighted in red and in the structural alignment (cluster 86…dark grey, cluster 40…grey) the residues of the GxxxGxxxT motif are highlighted for orientation. Additionally the distances between the glycine and threonine backbone atoms of the GxxxGxxxT interface is given for the structure of cluster 86. (D) The survival time (x-axis) of the NMR interface once it appeared in the simulation (either at the start or by reformation during simulation) is plotted against the probability of the presence of the NMR interface for all four membrane systems.

After establishing the model of the RBC membrane we investigated the dynamics of the protein. Initially we analyzed the stability of the NMR interface during the whole simulation time of 10 μs in the model of the RBC membrane and determined which rearrangements could be observed. We performed ten simulations of the whole system with different random seeds. Indeed, in all of the ten simulations a rearrangement of the NMR interface is observed (Fig 2A) and additional interfaces occur. The identification of different interfaces for GpA is not unexpected and was also observed by other approaches: a large dimer interface space for GpA was observed by a surface-based modeling approach [43] and several interfaces were predicted by Monte Carlo simulations to exist in a membrane mimicking environment [44].

As expected, the NMR interface (assigned in here as cluster 86;) occurs with a high frequency, which was expected because all simulations were started with this structure (Fig 2B). Interestingly, we observed a second interface in all ten simulations with high overall frequency (cluster 40; Fig 2B). Both interfaces occupy different sides of the helix and have different key resides which form the interface (Fig 2C). The interface of cluster 40 was observed with very low frequency in self-assembly simulations of 10 μs length in pure PC model membranes this interface ([40], S3 Fig). This prompted the question whether head groups or cholesterol have a major impact on interface structure and stability. Thus, we analyzed a system where all head groups were replaced by cholin (nFA+CHOL), a system without cholesterol (nFA+head) and a system without cholesterol and with only cholin head groups. The latter is a pure PC membrane with a native-like composition of fatty acids (nFA).

In contrast to the native membrane, where a reformation of NMR interface was only observed once after it was gone for at least 0.5 μs (Fig 2A), this event is more frequently observed in membranes missing cholesterol (S3 Fig). Indeed, in the various membranes different interface frequencies were observed. The adopted GpA dimer interfaces in the native-like membrane are most comparable to the interfaces of the nFA+CHOL membrane (Fig 2B; correlation: 0.80). Furthermore, the adopted interfaces are similar for both membranes missing cholesterol (nFA+head and nFA; Fig 2B; correlation: 0.95) and the most frequent interface (cluster 9) is also one of the most frequent interfaces in the self-assembly simulations in pure PC model membranes, which also lack cholesterol ([40], S3 Fig). These results indicate that cholesterol has a major impact on the preferred interface of the protein.

Next to the preferred interface, the stability of the NMR interface is influenced by cholesterol (Fig 2D). In the presence of cholesterol the time until the NMR interface rearranged into another interface is generally higher than in simulations without cholesterol (Fig 2D). In line it is also shown *in vitro* by FRET that the GpA dimer fraction is higher in PC model membranes containing cholesterol than in pure PC model membranes [45]. Thus, the simulations reproduce the biological behavior of the dimer. Whether this stabilization of the NMR interface is caused by a structural stabilization of cholesterol or whether it just reflects the slower lipid diffusion in membranes with cholesterol is not further investigated (2.05/2.43*10^−7^ cm^2^/s (native/nFA+CHOL) vs. 8.74/9.38*10^−7^ cm^2^/s (vs. nFA+head/nFA)).

### Protein-lipid interaction

**Fig 3:**
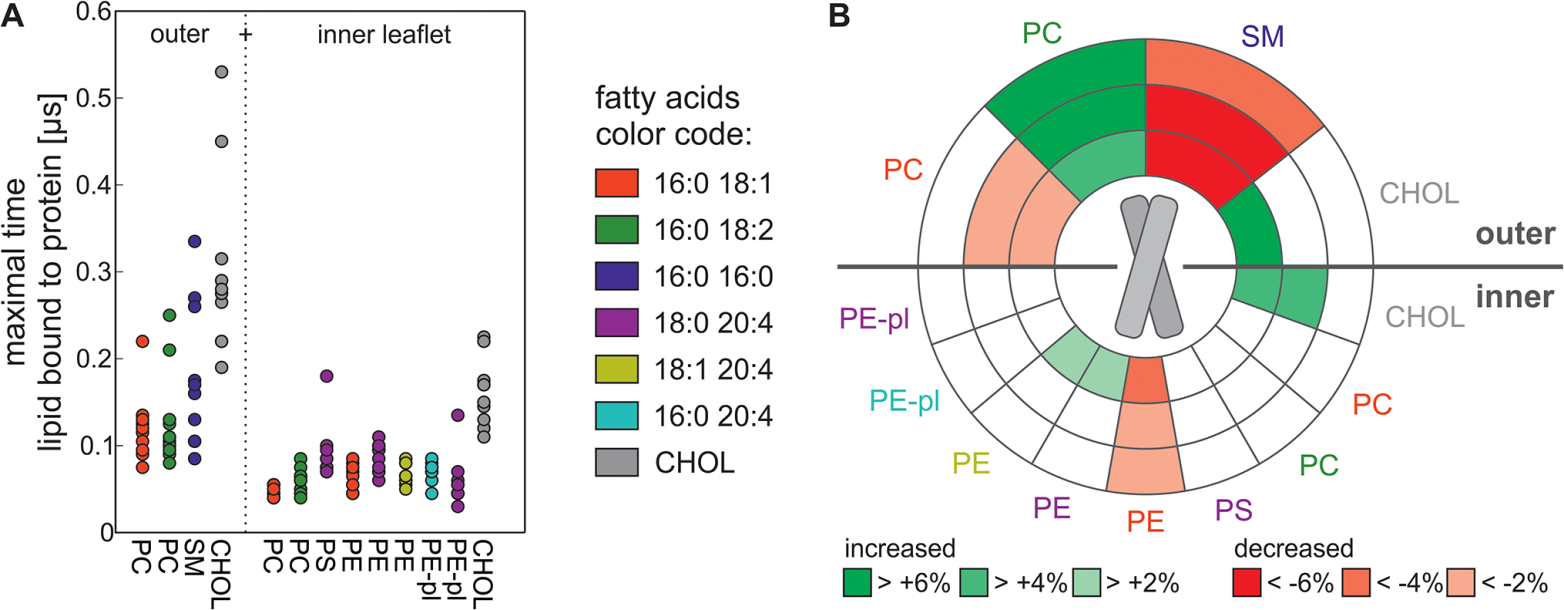
Interaction of lipids and protein in the RBC plasma membrane. (A) The maximal time (y-axis) a lipid is adjacent to the protein of each of the ten simulations is plotted for the different lipid species. The head group is given below, the fatty acid is color coded. Lipids of the outer and inner leaflet are shown separately. (B) Plotted are the first (distance < 0.658 nm), second (0.658 nm < distance < 0.918 nm) and third (0.918 nm < distance < 1.178 nm) hydration shell around the protein for the outer (upper part of the circle) and inner leaflet (lower part of the circle). The head group is given and the color of the label indicates the fatty acid. For each lipid it is given in color code, if its occurrence in the respective hydration shell is increased (green) or decreased (red) in comparison to the lipid frequency in the corresponding leaflet.

Focusing on the interaction of the protein with the lipids and cholesterol it becomes obvious that cholesterol is attached to the protein for the longest time when compared to other lipids of the leaflet (Fig 3A) and it is proven that these cholesterol molecules diffuse freely to the protein and are not attached to the protein at the beginning of the simulation (S5 Fig). Additionally, cholesterol is preferred in the first hydration shell around the protein on both leaflets (Fig 3B). This behavior is independent of the lipid head group distribution, because the same result is observed for the PC+CHOL membrane (S6 and S7 Figs). These findings are in good agreement to the experimental observation that in vesicles containing DMPC and cholesterol the latter is enriched in the vicinity of GpA [46,47]. Furthermore, the observed close vicinity of cholesterol to the transmembrane region fits to the influence of cholesterol on the dynamics of the protein as well (Fig 2). Analyzing which residues interact with lipids we noticed that the side chain beads of V84 and I88 interact nearly the whole simulation time (>95%) with cholesterol, a phenomenon which is not observed for other lipids and other residues of the protein. Interestingly both residues flank the GxxxGxxxT motif of the NMR interface and both residues are able to interact with one cholesterol molecule (S8 Fig), which is found often during the simulation. Both residues are not involved in the interface which is formed in cluster 40 (Fig 2C).

The association times of lipids with the protein do not only depend on the presence of cholesterol in the membrane, but also on the properties of the surrounding lipids of the corresponding leaflet. The time where cholesterol is adjacent to the protein is shorter in the inner leaflet than in the outer leaflet (Fig 3A). The same is observed for the nFA+CHOL membrane indicating that it is not the head group which enforces this behavior. The leaflet dependency is also seen for the phospholipids. For example PC present in the outer leaflet is longer in close vicinity to the transmembrane domain of the protein than PC in the inner leaflet (Fig 3A). Moreover, the observed enrichment of outer leaflet PC (16:0/18:2) in the hydration shell of the peptide is not observed for the PC in the inner leaflet (Fig 3B). Additionally the maximal time is generally less in the membranes missing cholesterol (S6 Fig). This suggests that the time a lipid is bound to the protein depends on cholesterol and the fatty acid composition of the corresponding leaflet, which in turn influences the membrane fluidity. Indeed is the phospholipid diffusion slower in the outer leaflet compared to the inner leaflet (S2 Table), which is most likely caused, be the higher amount of cholesterol. Furthermore the diffusion of lipids with polyunsaturated fatty acids is higher when compared to lipids with saturated or monounsaturated fatty acids (S2 Table). This suggests that it is possible that the different timescales observed for the interaction between the protein and the lipids are at least partially caused be the different diffusion rates.

In contrast to the interface formation of the GpA dimer, which was not dependent on the lipid head groups, we realized that the head groups influence the interaction time of the lipid with the protein. For example the experimentally determined preference of GpA for PS over PC [48] is reproduced by the simulation, because in the inner leaflet PS is longer bound to the protein than PC (Fig 3A). Furthermore, we observed a large difference in the maximal peptide binding time of SM (16:0/16:0) in the native-like membrane and of the corresponding DPPC in nFA+CHOL membrane. Here, DPPC shows a reduced maximal binding time to the protein (Fig 3A, S6 Fig) indicating that the head group (or to be more precise in this case: the lipid backbone) plays a. In addition, in the inner leaflet of the native-like membrane PE (16:0/18:1) shows a higher maximal association time with the protein than PC (16:0/18:1) (Fig 3A). Again, this parallels the experimental observation that GpA prefers the head group of PE over that of PC in unilamellar vesicles [49]. Interestingly, this tendency is not observed in the membrane missing cholesterol (nFA+head, S6 Fig). This indicates that both, the lipid head group and the presence of cholesterol, influence the interaction with the protein.

In support of the notion that the head group has an impact on the lipid association with the peptide, we did not observe an increased occurrence of lipids with the same fatty acid as PE while analyzing the RBC plasma membrane (Fig 3B). Particularly the charged PS lipid is neither preferred nor avoided in the hydration shell around the protein (Fig 3B). On the first thought this is surprising, because it was shown that GpA extracted from RBC membranes contains a significant amount of PS molecules and that GpA interacts strongly with monolayers consisting of PS lipids [50]. However, on the one hand, PS is highly abundant in the inner leaflet (Table 1) and thus, it still interacts with the peptide although the occurrence in the vicinity of the peptide is comparable to the average density in the membrane. On the other hand, the time PS is bound to the protein is the second longest for all inner leaflet lipids (Fig 3A), which is consistent with a high interaction between the lipid and the peptide. Nevertheless, the observed absence of an enrichment of PS in the neighborhood of the protein is consistent with the observed reduction of PS interaction with the GpA in the presence of PE lipids (1:1 mixture) and moreover, with the disruption of the interaction between GpA and PS when cholesterol (1:1) is present in the monolayer [50]. Therefore, our observation most likely reflects the native situation in the RBC plasma membrane. Moreover, this supports the notion that the head group has an impact on the interaction between lipid and protein.

As mentioned before, the protein prefers cholesterol in its first hydration shell. Moreover, it also prefers polyunsaturated lipids, and avoids saturated lipids. In the outer leaflet the protein avoids the saturated SM lipid and prefers a polyunsaturated PC lipid in its first three hydration shells (Fig 3B) Interestingly, this stands in contrast to the time where the single lipid is adjacent to the protein as SM is longer bound than to unsaturated PC lipids (Fig 3A) The situation for the inner leaflet is comparable, although it does not contain fully saturated lipids: PE lipids, which contain only one double bond (PE 16:0 18:1), are less frequent in the first three hydration shells of the protein. In contrast PE lipids with more double bonds are slightly enriched in the first hydration shell around the protein (Fig 3B). This shows that the fatty acid has a great impact on which lipid surrounds the protein, which is in agreement with NMR measurements demonstrating that GpA prefers unsaturated lipids over saturated lipids in PC membranes [51]. The situation in the other three membranes, (nFA+CHOL, nFA+head, nFA) is very similar to the native-like membrane. Saturated lipids are decreased in the first hydration shells around the protein and unsaturated lipids and cholesterol (if present) are increased (S7 Fig). This suggests that it is mainly the fatty acid, which influences which lipid surrounds the protein.

So far we conclude that both components of the lipids–the head group and the fatty acids-, the fatty acid composition of the leaflet and the cholesterol content influence the association of a lipid with the protein. The detailed analyses of the different analyzed membrane systems suggest a large impact of the fatty acids. However, we realized that lipids that interact longest with the protein are not necessarily lipids that are enriched in the hydration shell around the protein when compared to the global concentration in the membrane. It becomes obvious that the most abundant membrane components, which are cholesterol and SM in case of the outer leaflet, as well as cholesterol and PS in the inner leaflet, interact longest. In contrast, the enrichment in the hydration shell around the protein is lipid- and not lipid concentration-dependent (Fig 3B).

### Domain formation and protein sorting

In idealized plasma membranes domains with L_o_ and L_d_ character are formed during CG MD simulations [24]. On the one hand we show that the protein prefers unsaturated fatty acids in its hydration shell (Fig 3B), indicating that it is located in the L_d_-like domain. On the other hand, single cholesterol and saturated lipid molecules, which are typically enriched in the L_o_ domain, interact longer with GpA than polyunsaturated lipids (Fig 3A) Thus, we investigated whether L_o_ and L_d_ domains are formed in the model RBC plasma membrane with GpA dimer, and in case similar domains exist, in which domain the protein is located. This analysis was of particular interest, because the native-like RBC plasma membrane used in here has a higher cholesterol content than the previously published model [24].

**Fig 4:**
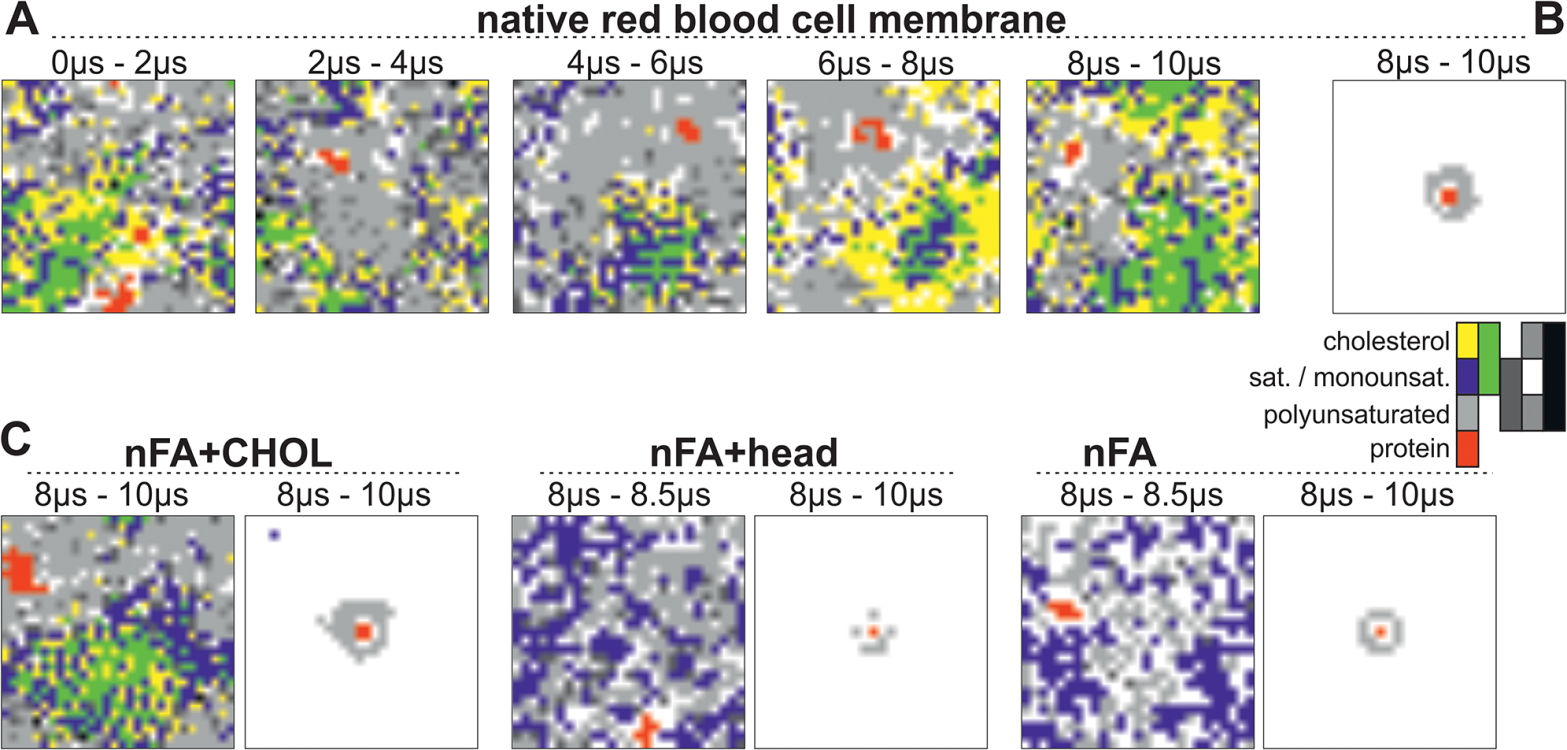
Domain formation and protein sorting in the RBC plasma membrane. (A) 2D density plot for one representative simulation of the RBC plasma membrane in the time course of the simulation, each plot corresponds to a 2 μs window. Areas where the protein is located are shown in red. Areas, which are enriched in cholesterol (yellow), saturated and monounsaturated lipids (blue) or both (green), form the L_o_-like domain. Areas, which are enriched in polyunsaturated lipids, but not in cholesterol or saturated and monounsaturated lipids, are shown in light grey and form the L_d_-like domain. A mix of all lipids is shown in black, a mix of cholesterol and polyunsaturated lipids is shown in grey and a mix of polyunsaturated and mono- and saturated lipids is shown in dark grey. Areas, where no lipid or protein is enriched, are white. (B) Density map calculated after overlay of the last 2 μs of all ten simulations with trajectories centered at the GpA dimer. The mix density maps for the single simulations are shown in S9 Fig (C) The 2D density plot (left, as in A) and the overlay of all ten simulations (right, as in B) for the nFA+CHOL, the nFA+head and the nFA membrane for the indicated time window is shown. The shorter time frame for membranes without cholesterol is used, because otherwise a nearly equal distribution of all lipids is observed.

As in the idealized plasma membrane [24], we also observe membrane areas enriched in cholesterol using the model of the RBC membrane. Furthermore, these areas are enriched in lipids with saturated and monounsaturated fatty acids as well, which demonstrates that membrane domains with L_o_-like character are formed (Fig 4) Consequently, the remaining part of the simulation box is enriched in lipids with polyunsaturated fatty acids. Thus, using the realistic cholesterol concentration of RBC membrane regions with L_d_ character are formed as well (Fig 4A, S9 Fig). Domain formation and protein sorting in all four membranes).

The L_o_ and L_d_ domains are dynamic and assemble and disassemble on the microsecond timescale and move across the whole simulation box. The protein also diffuses through the whole simulation box, but resides in the L_d_-like domain (Fig 4A and 4B; S4 Fig). This finding is in agreement with simulations of model membranes, which also show that model peptides are partitioned into the L_d_ phase during CG MD simulations [13]. The presence of different head groups in the membrane is not essential for the formation of areas, which are enriched in special fatty acids, as the same behavior is observed for the nFA+CHOL membrane (S9 Fig). This is in agreement with the observation that glycophorin A prefers the fluid phases in model membranes [52,53]. As expected, L_o_ and L_d_ domains are not observed in membranes without cholesterol (nFA+head and nFA). This confirms that cholesterol is necessary for the formation of the L_o_- and L_d_-ike domains in the RBC plasma membrane and is in agreement with results obtained from model membranes where cholesterol is essential for complete phase separation [54]. However, it is also important to note that no macroscopic phase separation occurs in the RBC plasma membrane as known from model membranes. In agreement, the area surrounding the protein which is enriched in saturated fatty acids is larger for the cholesterol-containing membranes in comparison to membranes without cholesterol (S9C Fig), indicating that in fact the protein prefers unsaturated fatty acids in its neighborhood even when domain formation does not occur in the absence of cholesterol.

## Conclusion

The behavior of the model of the RBC plasma membrane presented in here is in good agreement with experimental measurements for the RBC plasma membrane, available data for protein-protein and protein-lipid interactions therein and in model membranes. Taken together cholesterol has a great impact on the dynamics of the protein as well as on the preferred dimer interfaces, while the head group has only a minor but measureable influence. In agreement, cholesterol is also the crucial component for domain formation in the plasma membrane-containing saturated and unsaturated fatty acids. The model protein GpA prefers areas, which are enriched in lipids with unsaturated fatty acids, indicating that in general proteins without lipid anchor are sorted into these domains in the plasma membrane. Together with our previous results on the influence of different fatty acids on dimer formation [40] we conclude that fatty acids and the presence of cholesterol are the major determinants that influence protein interaction (and most likely function) in native membranes without having a direct catalytic or structural role for the corresponding protein. In model membranes a huge amount of protein can induce domain formation [54], whether this is the case in native membranes needs to be investigated in future. Furthermore, it will be interesting to investigate the behavior of proteins with gangliosides and lipid anchors in the native membrane, as it is shown for model membranes that these anchors can influence the sorting [14].

## Supporting Information

S1 Fig Comparison of ester and ether membranes containing cholesterol. (PDF)

S2 Fig Comparison of lipids surrounding the protein in the different simulations. (PDF)

S3 Fig Comparison of simulations started with the NMR dimer in membranes containing at least the native content of fatty acids to self-assembly simulations carried out in pure PC model membranes. (PDF)

S4 Fig Occurrence of the NMR interface in membranes with native composition of fatty acids. (PDF)

S5 Fig Distance between cholesterol and the protein. (PDF)

S6 Fig Maximal time a lipid is adjacent to the protein for the nFA+CHOL, nFA+head and nFA membrane. (PDF)

S7 Fig Hydration shells around the protein for the nFA+CHOL, nFA+head and nFA membrane. (PDF)

S8 Fig Interaction of the protein with cholesterol. (PDF)

S9 Fig Domain formation and protein sorting in all four membranes. (PDF)

S1 Table Composition of all membranes with native fatty acid composition. (PDF)

S2 Table Lipid diffusion rates of the native membrane. (PDF)
