# Supplementary material for: Dynamics of the Glycophorin A Dimer in Membranes of Native-Like Composition Uncovered by Coarse-Grained Molecular Dynamics Simulations
Source: PLoS One. 2015 Jul 29;10(7):e0133999. doi: 10.1371/journal.pone.0133999 (PMC4519189; doi:10.1371/journal.pone.0133999)
Supplement: S1 Fig — (PDF) [file pone.0133999.s001.pdf]

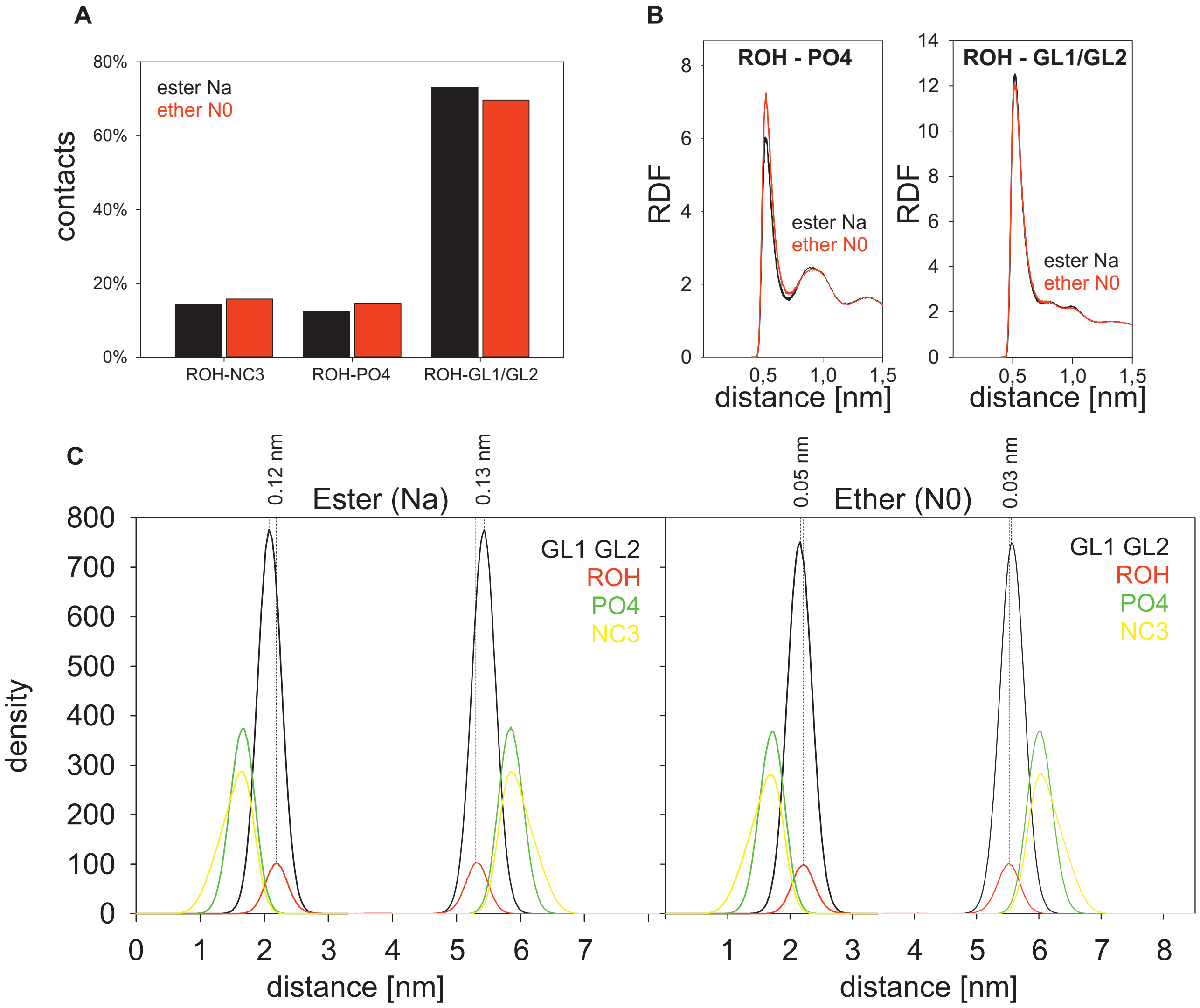

Figure S1. Comparison of ester and ether membranes containing cholesterol

A membrane containing the standard ester DPPC (128 molecules, linker: N_a type) lipids and cholesterol (32 molecules) and a membrane containing the corresponding ether lipid (DHPC, 128 molecules, linker: N_0 type) and cholesterol (32 molecules) are each simulated for 2 µs. **A.** For all cholesterol molecules interacting with a phospholipid it is counted which lipid head group bead was nearest to the ROH bead of cholesterol. One can see that the linker beads are less frequent the nearest bead when compared membranes with ester lipids and ether lipids. In line the PO4 and NH4 beads become more frequently the nearest bead for ether lipids. **B.** In the left panel the RDF between the ROH bead and linker bead of the phospholipid is shown and in the right panel the RDF between the ROH bead and the PO4 bead of the phospholipid is shown. **C.** The density profile of both membranes is shown and the distance between the GL1/GL2 and the ROH bead as extracted from the density profile is indicated, showing that cholesterol is located nearer to the water interface in the ether membrane.
