# Supplementary material for: Dynamics of the Glycophorin A Dimer in Membranes of Native-Like Composition Uncovered by Coarse-Grained Molecular Dynamics Simulations
Source: PLoS One. 2015 Jul 29;10(7):e0133999. doi: 10.1371/journal.pone.0133999 (PMC4519189; doi:10.1371/journal.pone.0133999)
Supplement: S2 Fig — (PDF) [file pone.0133999.s002.pdf]

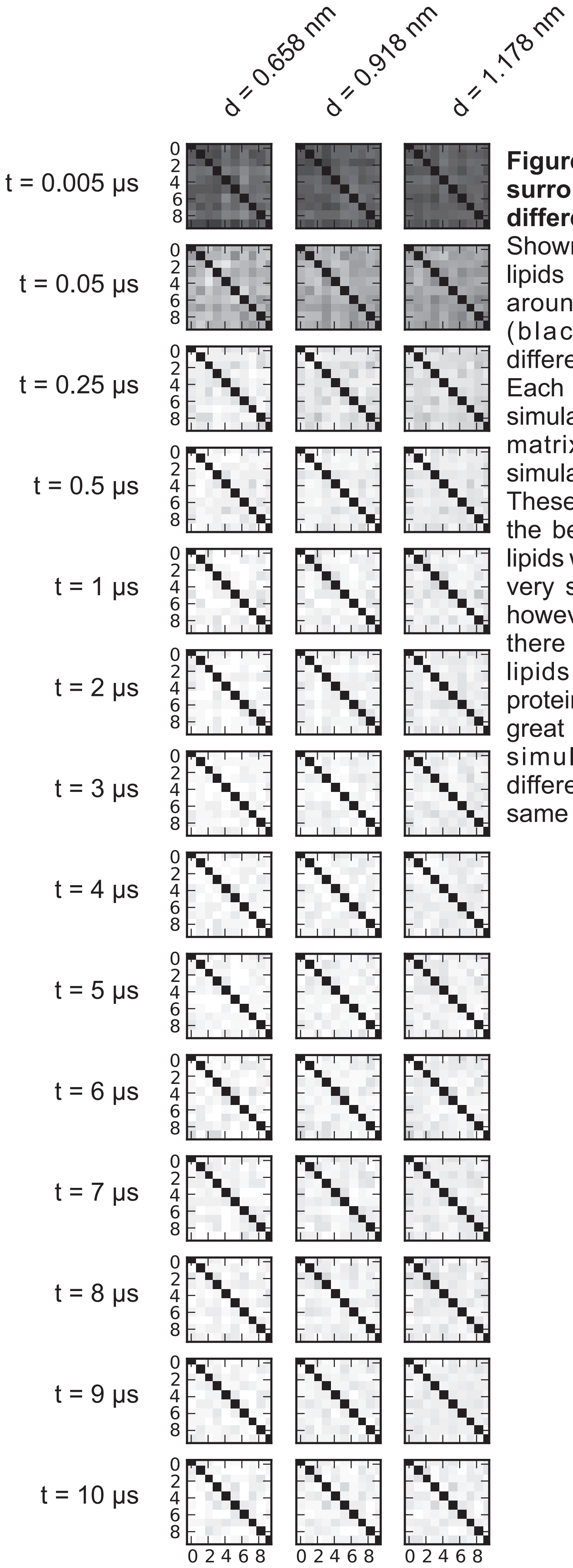

Figure S2. Comparison of lipids surrounding the protein in the different simulations

Shown is the percentage of identical lipids in the three hydration shells around the protein in grey scale (black…all identic; white…all different) for different time points. Each field correspond to a pair of simulations, in the diagonal of the matrix the similarity to the own simulation is plotted.

These plots show that shortly after the beginning of the simulation, the lipids which surrounds the protein are very similar for the ten simulations, however already after 0.25 to 0.5 µs there are nearly completely other lipids in the environment of the protein indicating that it has not a great influence to the outcome if the simulations were started from different random insertions or the same insertion as done in here.
