# Supplementary material for: Dynamics of the Glycophorin A Dimer in Membranes of Native-Like Composition Uncovered by Coarse-Grained Molecular Dynamics Simulations
Source: PLoS One. 2015 Jul 29;10(7):e0133999. doi: 10.1371/journal.pone.0133999 (PMC4519189; doi:10.1371/journal.pone.0133999)
Supplement: S3 Fig — (PDF) [file pone.0133999.s003.pdf]

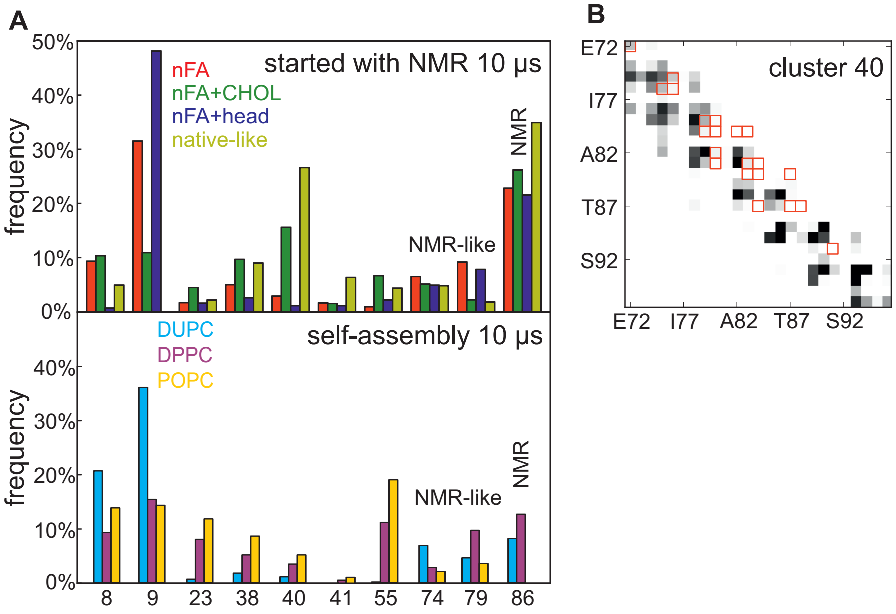

Figure S3. Comparison of simulations started with the NMR dimer in membranes containing at least the native content of fatty acids to self-assembly simulations carried out in pure PC model membranes

A. The simulations described in Flinner *et al.* 2014 were clustered together with the simulations described in here in order to assign identical cluster numbers for the same interface for both data sets. In the upper panel the frequencies shown in Figure 2B are redrawn for better comparison to frequencies shown in the lower panel. This lower panel contains the frequencies of the long self-assembly simulations described in Flinner *et al.* 2014 carried out in DUPC, DPPC and POPC model membranes. B. The contact plot for the interfaces of cluster 40 is shown, which is enriched in the cholesterol containing membranes. In general the frequent clusters mentioned in Flinner *et al.* 2014 correspond as follows to the clusters of the new clustering for this manuscript: 79 (old) = 8 (new), 81 = 9, 52 = 55, 14 = 86 (NMR), 12 = 79 (NMR-like), 16 = 74 (NMR-like).
