# Supplementary material for: Dynamics of the Glycophorin A Dimer in Membranes of Native-Like Composition Uncovered by Coarse-Grained Molecular Dynamics Simulations
Source: PLoS One. 2015 Jul 29;10(7):e0133999. doi: 10.1371/journal.pone.0133999 (PMC4519189; doi:10.1371/journal.pone.0133999)
Supplement: S4 Fig — (PDF) [file pone.0133999.s004.pdf]

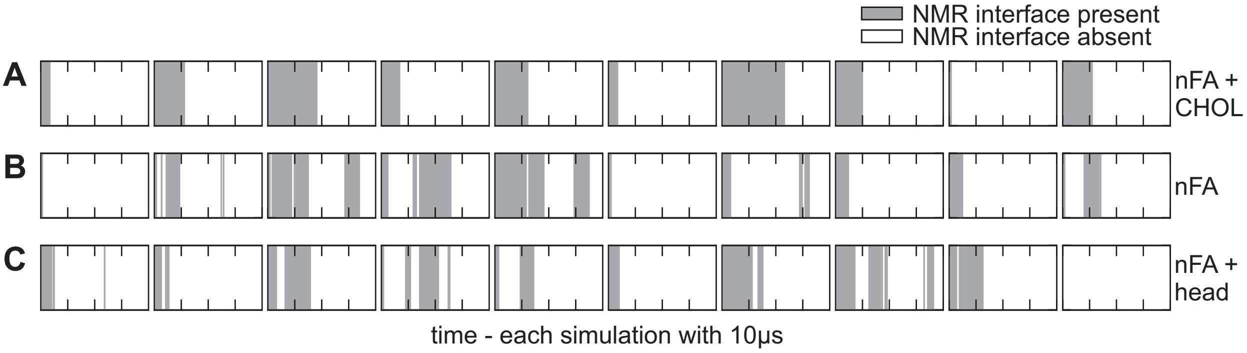

Figure S4. Occurrence of the NMR interface in membranes with native composition of fatty acids

For all ten simulations performed in the membrane containing **A.** a native composition of fatty acids fused to the PC head group and cholesterol (nFA+CHOL) **B.** a native composition of fatty acids fused to the PC head group but missing cholesterol (nFA) **C.** a native composition of fatty acids fused to native head groups (nFA+head) the presences of the NMR interface is plotted for each time point in grey.
