# Supplementary material for: Dynamics of the Glycophorin A Dimer in Membranes of Native-Like Composition Uncovered by Coarse-Grained Molecular Dynamics Simulations
Source: PLoS One. 2015 Jul 29;10(7):e0133999. doi: 10.1371/journal.pone.0133999 (PMC4519189; doi:10.1371/journal.pone.0133999)
Supplement: S5 Fig — (PDF) [file pone.0133999.s005.pdf]

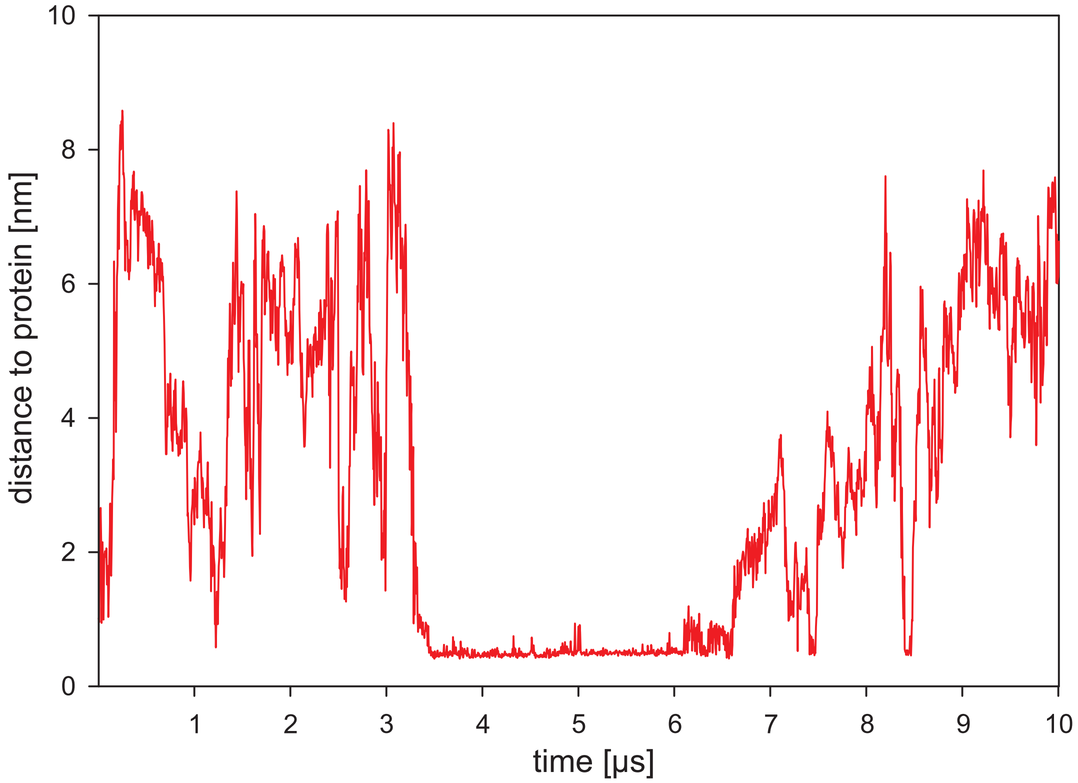

Figure S5. Distance between cholesterol and the protein
The distance of the cholesterol molecule which is neighbored longest to the protein (red) is plotted against the simulation time.
