# Supplementary material for: Dynamics of the Glycophorin A Dimer in Membranes of Native-Like Composition Uncovered by Coarse-Grained Molecular Dynamics Simulations
Source: PLoS One. 2015 Jul 29;10(7):e0133999. doi: 10.1371/journal.pone.0133999 (PMC4519189; doi:10.1371/journal.pone.0133999)
Supplement: S6 Fig — (PDF) [file pone.0133999.s006.pdf]

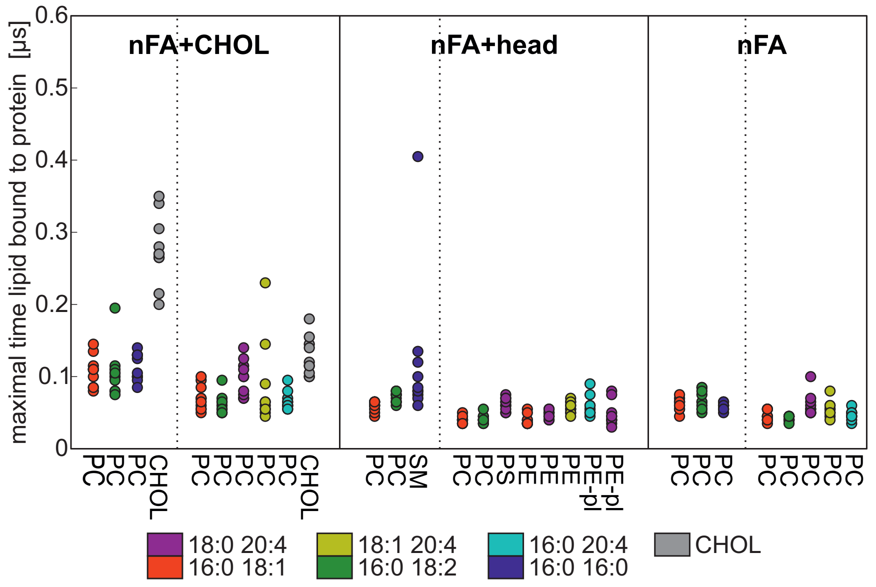

Figure S6. Maximal time a lipid is adjacent to the protein for the nFA+CHOL, nFA+head and nFA membrane

On the y-axis the maximal time a lipid is found to be adjacent to the protein is plotted for the different lipid species. The head group is given below, the fatty acid is color coded. Lipids of the outer leaflet are shown left, the lipids of the inner leaflet are shown right. Each dot corresponds to the maximal value of one of the ten simulations carried out inthe nFA+CHOL, nFA+head or nFA membrane.
