# Supplementary material for: Dynamics of the Glycophorin A Dimer in Membranes of Native-Like Composition Uncovered by Coarse-Grained Molecular Dynamics Simulations
Source: PLoS One. 2015 Jul 29;10(7):e0133999. doi: 10.1371/journal.pone.0133999 (PMC4519189; doi:10.1371/journal.pone.0133999)
Supplement: S7 Fig — (PDF) [file pone.0133999.s007.pdf]

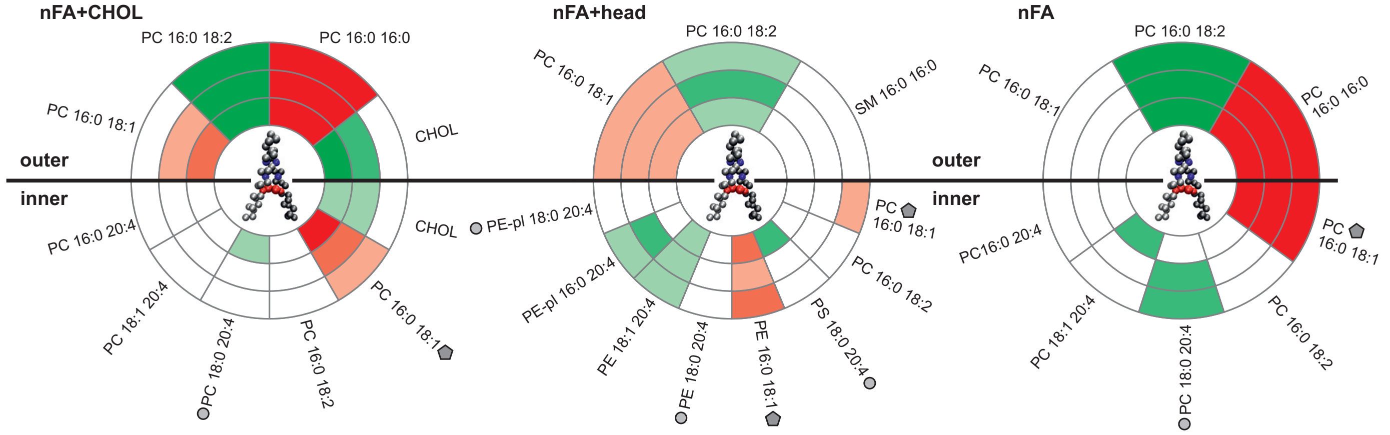

Figure S7. Hydration shells around the protein for the nFA+CHOL, nFA+head and nFA membrane.

Plotted are the first (distance < 0.658 nm), second (distance < 0.918 nm) and third (distance < 1.178 nm) hydration shell around the protein for the outer (upper part of the circle) and inner leaflet (lower part of the circle) for the nFA+CHOL, nFA+head and nFA membrane. Lipids with the same fatty acid, but different head groups are marked by a sign for clarity. For each lipid it is given in color code, if its occurrence in the respective hydration shell is increased (green) or decreased (red) in comparison to the lipid frequency in the corresponding leaflet.
