# Supplementary material for: Dynamics of the Glycophorin A Dimer in Membranes of Native-Like Composition Uncovered by Coarse-Grained Molecular Dynamics Simulations
Source: PLoS One. 2015 Jul 29;10(7):e0133999. doi: 10.1371/journal.pone.0133999 (PMC4519189; doi:10.1371/journal.pone.0133999)
Supplement: S8 Fig — (PDF) [file pone.0133999.s008.pdf]

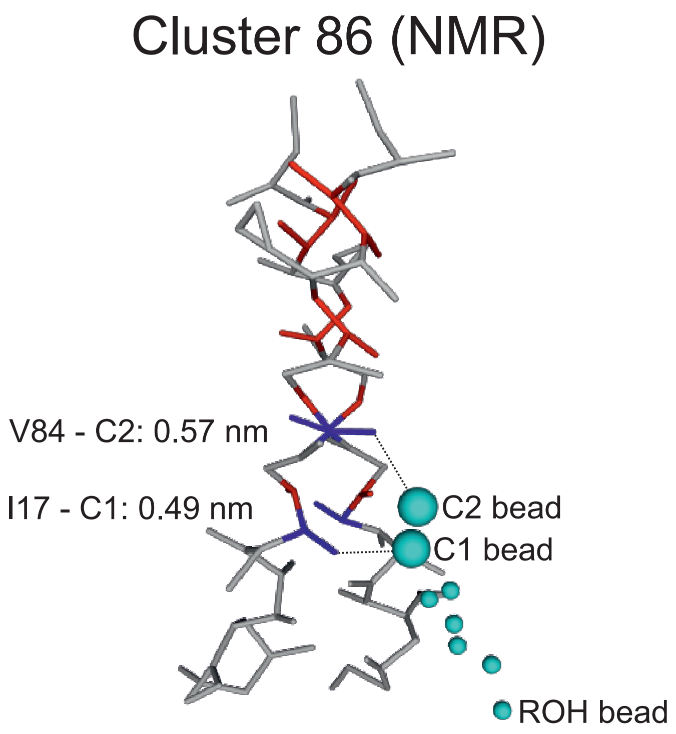

Fgure S8. Interaction of the protein with cholesterol
Shown is the representative structure of cluster 86 (NMR interface) in stick representations. Additionally the beads of a cholesterol molecule which interacts with residue V84 and I88 is shown. In general the residues interact most of the time with a cholesterol molecule and it is often observed that it interacts with the C1 and C2 bead of cholesterol, which model the carbon atoms 20-27 of cholesterol.
