# Supplementary material for: Dynamics of the Glycophorin A Dimer in Membranes of Native-Like Composition Uncovered by Coarse-Grained Molecular Dynamics Simulations
Source: PLoS One. 2015 Jul 29;10(7):e0133999. doi: 10.1371/journal.pone.0133999 (PMC4519189; doi:10.1371/journal.pone.0133999)
Supplement: S9 Fig — (PDF) [file pone.0133999.s009.pdf]

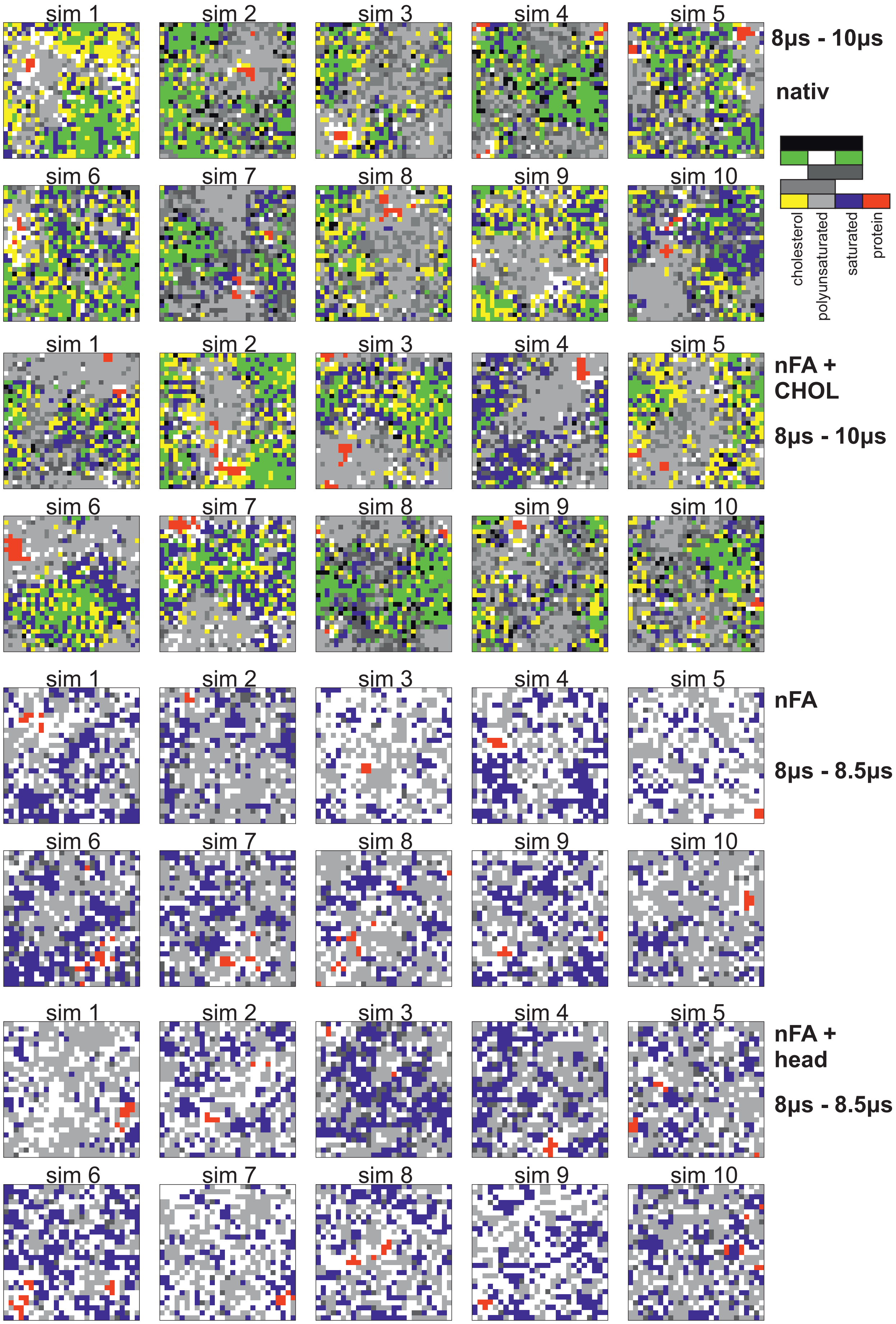

Figure S9. Domain formation and protein sorting in all four membranes
2D density plot for the indicated time window is shown for each of the ten simulations for the four different membrane systems. Areas where the protein is located are shown in red. Areas which were enriched in cholesterol (yellow), saturated and monounsaturated lipids (blue) or both (green) form the Lo-like domain. Areas which are enriched in polyunsaturated lipids, but not in cholesterol or saturated and monounsaturated lipids, are shown in light grey and form the Ld-like domain. A mix of all lipids is shown in black, a mix of cholesterol and polyunsaturated lipids is shown in grey and a mix of polyunsaturated and mono- and saturated lipids is shown in dark grey. Areas where no lipid or protein is enriched are white.
