# Supplementary material for: Dynamics of the Glycophorin A Dimer in Membranes of Native-Like Composition Uncovered by Coarse-Grained Molecular Dynamics Simulations
Source: PLoS One. 2015 Jul 29;10(7):e0133999. doi: 10.1371/journal.pone.0133999 (PMC4519189; doi:10.1371/journal.pone.0133999)
Supplement: S1 Table — (PDF) [file pone.0133999.s010.pdf]

Table S1. Composition of all membranes with native fatty acid composition

Exact composition for the native membrane and the systems derived from the native membrane, namely the membrane missing cholesterol (nFA+head), containing only the PC head group (nFA+CHOL) and containing only the PC head group and missing cholesterol (nFA) at the beginning of the simulation. The "atomistic" column gives the fatty acid for the corresponding lipid, in "CG" the CG representation is given (first number: number of beads; second number: number of beads representing double bonds)

lipid	fatty acid		native		nFA+head		nFA+CHOL		nFA	
	atomistic	CG	count inner	count outer	count inner	count outer	count inner	count outer	count inner	count outer
CHOL	-		245	245	-		245	245	-	
PC	16:0/18:2	4:0/4:2	25	63	25	63	25	63	25	63
	16:0/18:1	4:0/5:1	20	52	20	52	50	52	50	52
	16:0/16:0	4:0/4:0	-	-	-	-	-	140	-	140
	18:0/20:4	5:0/5:4					132	-	132	-
	18:1/20:4	5:1/5:4					20	-	20	-
	16:0/20:4	4:0/5:4					28	-	28	-
SM	16:0/16:0	4:0/4:0	-	140	-	140	-		-	
PS	18:0/20:4	5:0/5:4	90	-	90	-	-		-	
PE	16:0/18:1	4:0/4:2	30	-	30	-	-		-	
	18:0/20:4	5:0/5:4	30		30					
	18:1/20:4	5:1/5:4	20		20					
PE-pl	16:0/20:4	4:0/5:4	28	-	28	-	-		-	
	18:0/20:4	5:0/5:4	12		12					
			1,000 lipids<br>9,910 CG water<br>90 ions (Na+)		510 lipids<br>9,910 CG water<br>90 ions (Na+)		1,000 lipids<br>10,000 CG water		510 lipids<br>10,000 CG water	
