# Supplementary material for: Dynamics of the Glycophorin A Dimer in Membranes of Native-Like Composition Uncovered by Coarse-Grained Molecular Dynamics Simulations
Source: PLoS One. 2015 Jul 29;10(7):e0133999. doi: 10.1371/journal.pone.0133999 (PMC4519189; doi:10.1371/journal.pone.0133999)
Supplement: S2 Table — (PDF) [file pone.0133999.s011.pdf]

Table S2. Lipid diffusion rates of the native membrane

outer leaflet		inner leaflet	
**lipid**	**diffusion**	**lipid**	**diffusion**
SM 16:0/16:0	$0.67*10^{-7}$ cm^2/s	PE 16:0 18:1	$2.58*10^{-7}$ cm^2/s
PC 16:0/18:1	$0.78*10^{-7}$ cm^2/s	PE 18:0 20:4	$3.17*10^{-7}$ cm^2/s
PC 16:0/18:2	$1.04*10^{-7}$ cm^2/s	PE 18:1 20:4	$3.26*10^{-7}$ cm^2/s
		PEpl 16:0 20:4	$3.48*10^{-7}$ cm^2/s
		PEpl 18:0 20:4	$3.53*10^{-7}$ cm^2/s
		PS 18:0 20:4	$3.26*10^{-7}$ cm^2/s
		PC 16:0/18:1	$2.72*10^{-7}$ cm^2/s
		PC 16:0/18:2	$3.52*10^{-7}$ cm^2/s
cholesterol $2.69*10^{-7}$ cm^2/s			
